# Transcriptional Profiling of MEq-Dependent Genes in Marek’s Disease Resistant and Susceptible Inbred Chicken Lines

**DOI:** 10.1371/journal.pone.0078171

**Published:** 2013-10-21

**Authors:** Sugalesini Subramaniam, Likit Preeyanon, Hans H. Cheng

**Affiliations:** 1 Comparative Medicine and Integrative Biology Program, College of Veterinary Medicine, Michigan State University, East Lansing, Michigan, United States of America; 2 United States Department of Agriculture, Agricultural Research Service, Avian Disease and Oncology Laboratory, East Lansing, Michigan, United States of America; 3 Department of Microbiology and Molecular Genetics, Michigan State University, East Lansing, Michigan, United States of America; University of California, Davis, United States of America

## Abstract

Marek’s disease (MD) is an economically significant disease in chickens caused by the highly oncogenic Marek’s disease virus (MDV). Understanding the genes and biological pathways that confer MD genetic resistance should lead towards the development of more disease resistant commercial poultry flocks or improved MD vaccines. MDV mEq, a bZIP transcription factor, is largely attributed to viral oncogenicity though only a few host target genes have been described, which has impeded our understanding of MDV-induced tumorigenesis. Given the importance of mEq in MDV-induced pathogenesis, we explored the role of mEq in genetic resistance to MDV. Using global transcriptome analysis and cells from MD resistant or susceptible birds, we compared the response to infection with either wild type MDV or a nononcogenic recombinant lacking mEq. As a result, we identified a number of specific genes and pathways associated with either MD resistance or susceptibility. Additionally, integrating prior information from ChIP-seq, microarray analysis, and SNPs exhibiting allele-specific expression (ASE) in response to MDV infection, we were able to provide evidence for 24 genes that are polymorphic within mEq binding sites are likely to account for gene expression in an allele-specific manner and potentially for the underlying genetic differences in MD incidence.

## Introduction

Marek’s disease (MD) is a lymphoproliferative disease of poultry caused by Marek’s disease virus (MDV, *Gallid herpesvirus* 2), an oncogenic alphaherpesvirus. One of the main MD control strategies is vaccination. While MD vaccination reduces the incidence of tumor formation, it is not sterilizing, thus, does not prevent MDV from replicating or spreading amongst vaccinated birds. Additionally, field strains continue to evolve, with increased virulence in vaccinated birds. Losses with MD are further enhanced by the unpredictable and spontaneous outbreaks that occur even in vaccinated flocks [1,2]. Given the problems with vaccination, there is a need to pursue other strategies to combat MD. Identifying chickens with enhanced genetic resistance to MD is an attractive alternative to augment vaccinal control. Using genomic tools to identify genetic markers associated with MD resistance would be highly beneficial to select birds with superior disease resistance. A better understanding of the mechanisms of genetic resistance to MD would therefore contribute toward improved strategies to control the disease.

Currently, Avian Disease and Oncology Laboratory (ADOL) chicken lines 6 (MD resistant) and 7 (MD susceptible) have been developed to study the mechanisms underlying genetic resistance to MD [3-5]. These highly inbred (over 99%) White Leghorn lines share the same MHC haplotype, a genetic locus that has been shown to have a large effect on MD incidence [6]. Therefore, these lines enable us to focus on the remaining non-MHC genes that individually are smaller in effect size but cumulatively account for the majority of MD genetic resistance.

Selection for MD resistance is based on identifying genes where variation in their alleles is associated with variation in disease incidence. In our lab, two broad strategies have been employed and integrated. Namely, genome-wide genetic screens (e.g., QTL scans) to identify regions in the chicken genome containing the gene(s) of interest, and functional genomic screens (e.g., transcript profiling or virus-host protein-protein interaction screens) to provide candidate genes (reviewed in 7). Despite identifying three MD resistance genes (growth hormone, SCA2, and MHC class II [8-10] and many more strong candidates, like other complex traits, it has been very difficult to comprehensively identify the remaining genes that are involved in resistance.

Transcriptome analysis using spleens from MD resistant and susceptible lines identified several candidate genes related to resistance and susceptibility, most of which were related to the immune response [11,12]. There are differences in the proportion of CD4 and CD8 T cells between MD resistant and susceptible lines [13], and higher expression of immunoglobulin genes in MD resistant lines when compared to susceptible lines [14]. However, these studies have not specifically examined the influence of MDV Meq, a bZIP transcription factor and the viral oncogene, and its role on genetic resistance. Using global transcriptome analysis, we identified a number of genes and pathways that are consistently associated with MD resistance or susceptibility. We also show that heterozygous SNP sites in Meq-binding sites between lines 6 and 7 are associated with allele-specific expression, which may provide a mechanism that accounts for a proportion of the variation in MD genetic resistance between these two bird lines.

## Materials and Methods

### Cells and culture conditions

Chicken embryo fibroblasts (CEF) from day 10 embryos were prepared from ADOL specific pathogen free line 6 and line 7, and secondary cultures plated at a density of 10^7^ cells per 100 mm dish. Cells were cultured in Leibowitz’s L-15 and McCoy 5A media with 15% heat inactivated fetal bovine serum, 100 U of penicillin per ml, and maintained at 37 °C. In each of two experimental replicates, three plates each that were confluent for 24 h were infected with 10^4^ pfu of MDV derived from either Md5B40BAC1, our BAC clone that contains the Md5 strain MDV genome and generates virulent MDV, which we referred to as Md5 [15], or a derived nononcogenic recombinant from Md5B40BAC1 that lacks both copies of Meq, developed through recombineering [16], which we referred to as Md5**∆**Meq. For uninfected controls, an equal amount of uninfected CEF were added. Total RNA was isolated at 24, 48, and 96 hr from CEFs infected with either Md5 or Md5∆Meq as well as uninfected CEFs as controls.

### RNA extraction microarray procedure and data analysis

Total RNA for microarray hybridization was extracted using Absolutely RNA Miniprep Kit (Stratagene, Clara, CA, USA) according to the manufacturer’s instructions. RNA concentration was assessed using a Nanodrop ND-1000 spectrophotometer (Thermo Scientific, Wilmington, DE, USA) and the RNA integrity was determined using Agilent 2100 Bioanalyzer with a RNA 6000 Nano/Pico Assay (Agilent Technologies, Palo Alto, CA). Affymetrix GeneChip Chicken Genome Arrays (Affymetrix, Santa Clara, CA, USA) were used for microarray hybridization and data collection; this chip has probe sets for annotated chicken transcripts including all 17,179 chicken unigenes and Ensembl predicted genes. RNA preparation, hybridization and scanning were performed following protocols recommended by Affymetrix by the Michigan State University Research Technology Support Facility (rtsf.msu.edu). CEL files were generated that contained the summary intensities for each probe; NCBI accession no. GSE48454. The raw data files were loaded using the Affymetrix package, and the probe intensities and normalization were done using LIMMA (linear models for microarray data) [17]. The expression value of each probe set was normalized and calibrated using the open source 'R' statistical software (version 2.11.1) through the Bioconductor project (bioconductor.org). Pair wise comparisons between the groups including Md5 vs Md5**∆**Meq were performed by Fishers Least Significant Difference (LSD).

### Quantitative RT-PCR analysis

Total RNA was extracted using an RNeasy Mini Kit (Qiagen, Valencia, CA, USA). Complementary DNA was synthesized using a Superscript III First-Strand Synthesis System (Invitrogen, Carlsbad, CA, USA). Gene expression levels were measured using SYBR Green PCR Master Mix (Invitrogen) on an ABI 7500 Real-Time PCR System (Applied Biosystems, Foster City, CA, USA). The β-actin gene was used for normalization and each target gene was analyzed in triplicate. The PCR conditions were as follows: 95 °C for 10 min, followed by 40 cycles of 95 °C for 15 sec, 60 °C for 1 min, and 95 °C for 15 sec. At the end of amplification, a melting curve analysis was done by heating the PCR products to 65–95 °C, which was held for 15 sec at increments of 0.2 °C to measure fluorescence and confirm the presence of a single amplification product. For negative controls, no-RT was used as template in place of single-stranded cDNA in the qRT-PCR. The data analysis was performed with the comparative ∆∆Ct relative quantiﬁcation method [18].

### Pathway analysis

The differentially expressed genes (DEGs) were identified amongst uninfected, Md5-infected, and Md5∆Meq-infected lines 6 or 7 CEF. These genes were further analyzed for inclusion in Gene Ontology (GO) categories and pathways in order to examine their biological processes. Categorization of genes based on significant biological properties was done using Gene Ontology Project (http://www.geneontology.org/). The genes were grouped categories based on common biological properties.

The pathway analysis was carried out using Ingenuity Pathway Analysis software (IPA, Ingenuity Systems, Redwood City, CA, USA). The annotated genes were grouped into networks, functions, or canonical pathways. The data with gene IDs and expression fold-change were uploaded into the software. The gene IDs were mapped into its corresponding gene object in the Ingenuity Knowledge Base (IKB). The network of focus genes were generated based on the information contained in the IKB into a global molecular network. Functional analysis generates biological functions that are significant to the genes in the data uploaded. The canonical pathways are generated based on the DEGs from the data. All the IPA analyses were carried out based on Fisher’s exact test to determine the association between the differentially-regulated genes and the network, biological function, and canonical pathway.

## Results

### Identification of Meq-dependent genes related to genetic resistance and susceptibility

To determine the role of Meq in MD genetic resistance, we performed global transcriptome analysis in CEFs from lines 6 and 7, two highly inbred experimental White Leghorn chicken lines that differ greatly in MD incidence. To determine if genes in Meq-regulated pathways are influenced by the genetic resistance status of the host, the CEFs from both chicken lines were infected with either Md5, which is a fully virulent MDV, or Md5**∆**Meq, a recombinant MDV that lacks both copies of Meq and is nonocogenic. We chose three time points: 24, 48 and 96 hr to study the gene expression changes induced during these phases of virus infection. We hypothesized genes that are uniquely differentially expressed in Md5-infected groups and not in Md5**∆**Meq-infected cells indicate ones that are directly or indirectly dependent on Meq expression.

**Figure 1:**
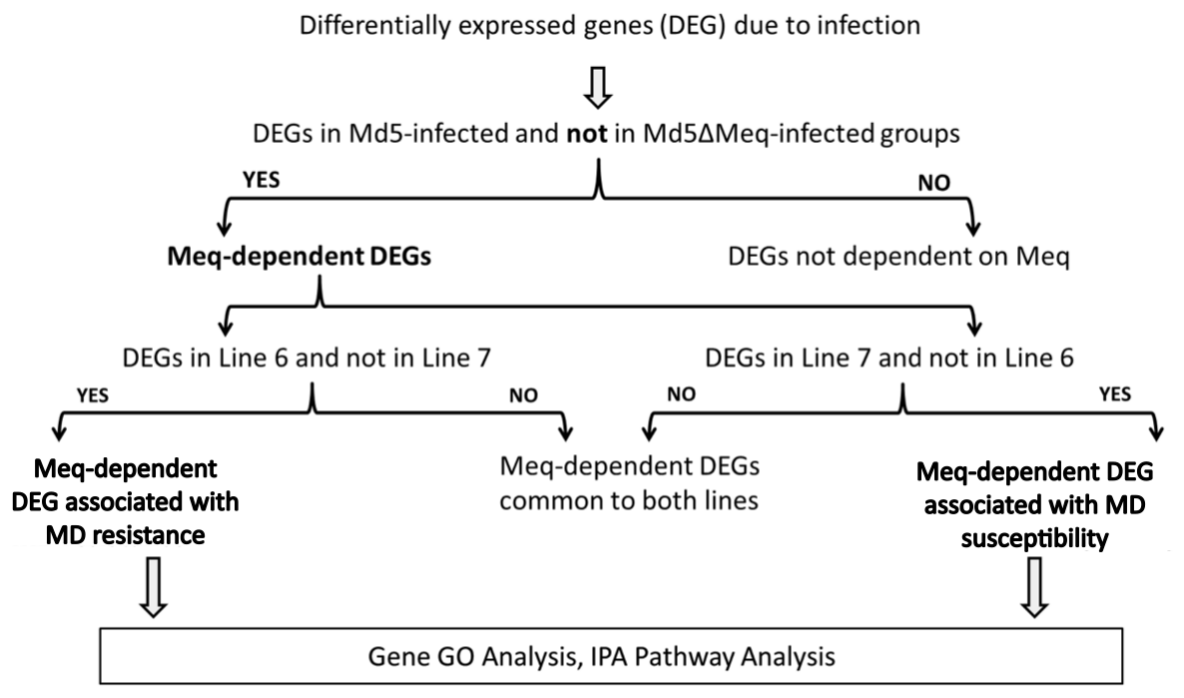
Schematic for analysis of experimental groups. The differentially expressed genes (DEGs) between Md5-infected and Md5**∆**Meq-infected groups compared to untreated control CEFs were obtained. Then DEGs present in Md5-infected group and not in Md5**∆**Meq-infected group were designated as Meq-dependent DEGs. These were further divided into DEGs specific to lines 6 or 7, and this set was used for GO categorization and IPA Pathway analyses.

**Figure 2:**
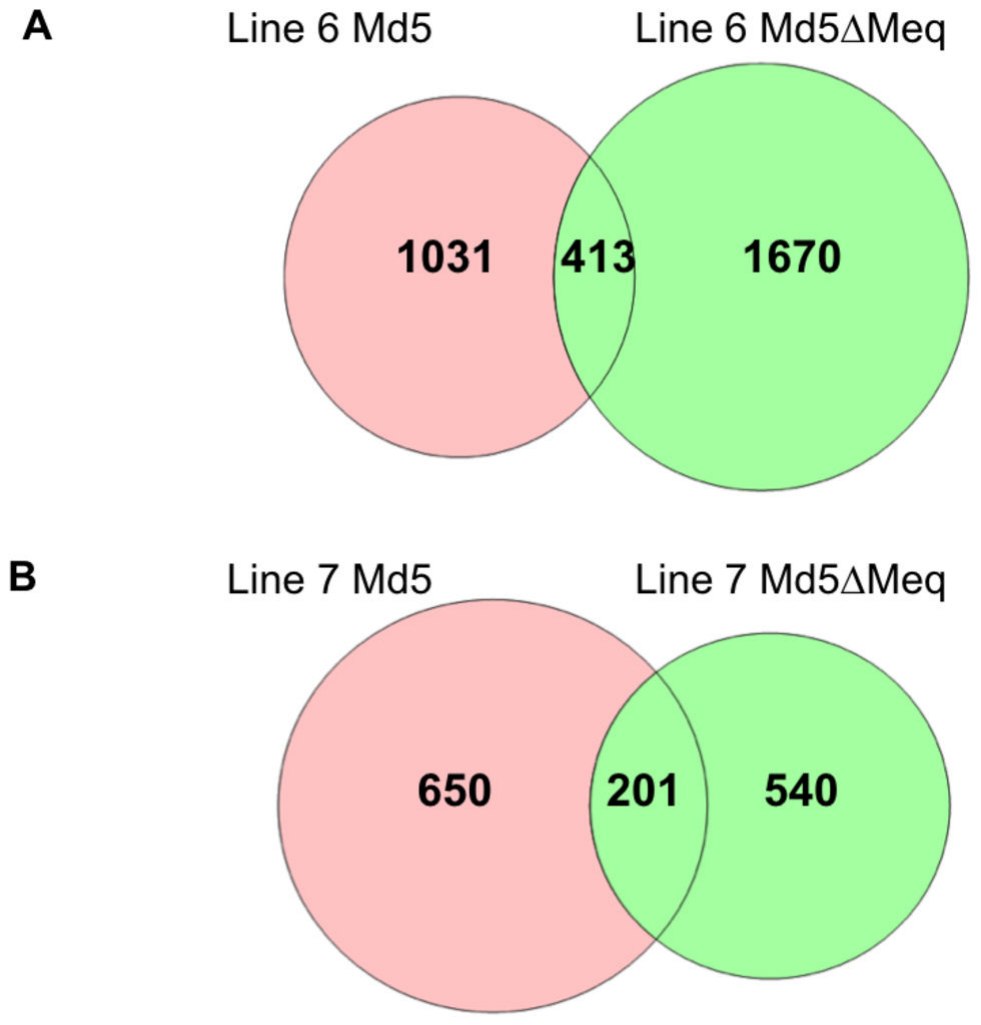
Identification of Meq-dependent genes in MD resistant or susceptible chicken lines. Venn diagram of comparison between two virus-infected groups in line 6 and line 7. Each circle depicts the number of differentially expressed genes compared to uninfected controls. The portion highlighted in pink in each diagram denotes the Meq-dependent genes.

**Figure 3:**
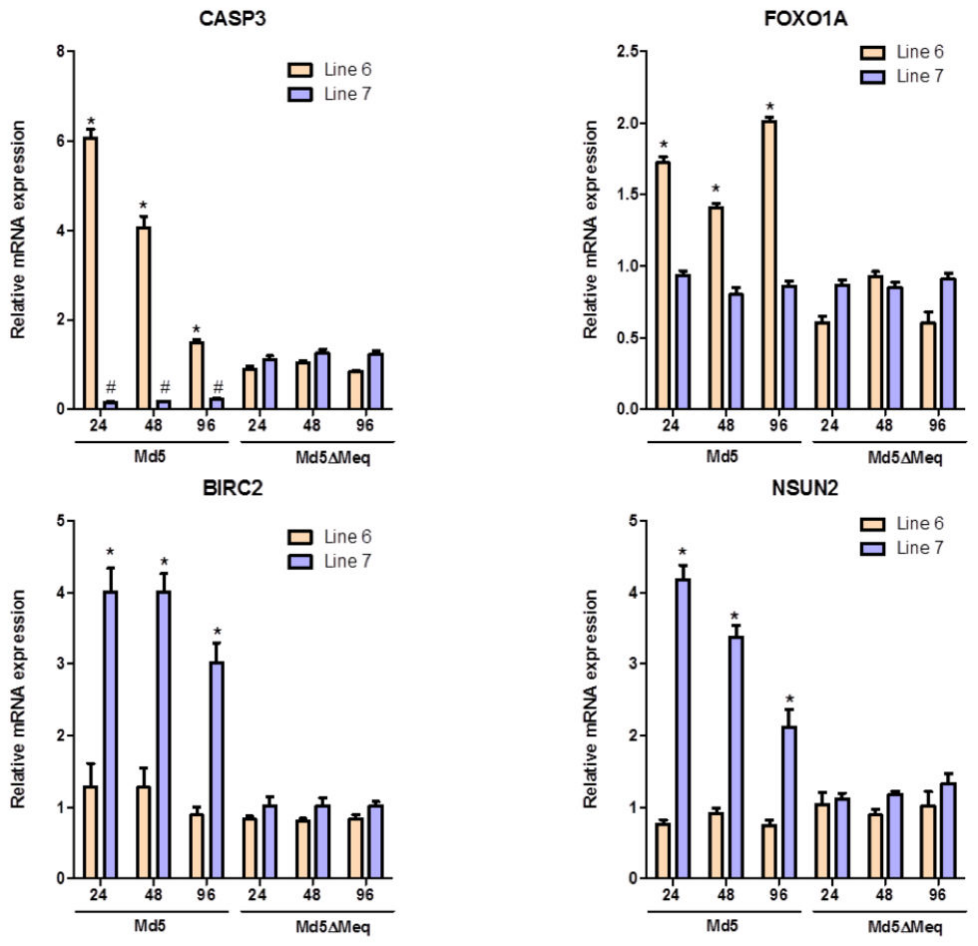
qPCR validation of microarray results. Validation by RT-qPCR of the microarray-based differentially expressed genes between line 6 and line 7 CEFs. Beta-actin was used as internal control. *P<0.05 compared to uninfected CEF as controls.

We made pair-wise comparisons to generate a list of genes that are unique to lines 6 and 7, which were further classified into genes dependent or not dependent on the expression of Meq as shown in (Figure 1). The details on DEGs at each time point in Md5-infected and Md5**∆**Meq-infected groups for each line are provided in Table 1. GO categorization for pathway analysis of lines 6 and 7 were performed using a ‘union’ of DEGs in all three time points. The number of Meq-dependent genes that are associated with MD resistance and susceptibility are shown in Figure 2A and 2B, respectively. The DEGs detected by microarrays were validated by qRT-PCR for a randomly subset of genes that were selected from the top biological networks. The fold-change as determined by qRT-PCR was significantly correlated to the findings from microarray data (r^2^=0.67; P<0.05) (Figure 3).

**Table 1:** Summary of differentially expressed genes in lines 6 and 7 using infection with virulent Md5 or avirulent Md5∆Meq.

	Line 6				Line 7			
	Md5		Md5**∆**Meq		Md5		Md5**∆**Meq	
	Up	Down	Up	Down	Up	Down	Up	Down
24 hrs	1075	346	1397	463	574	434	404	326
48 hrs	968	635	1167	883	438	375	1219	261
96 hrs	453	331	1204	266	618	106	97	393

**Figure 4:**
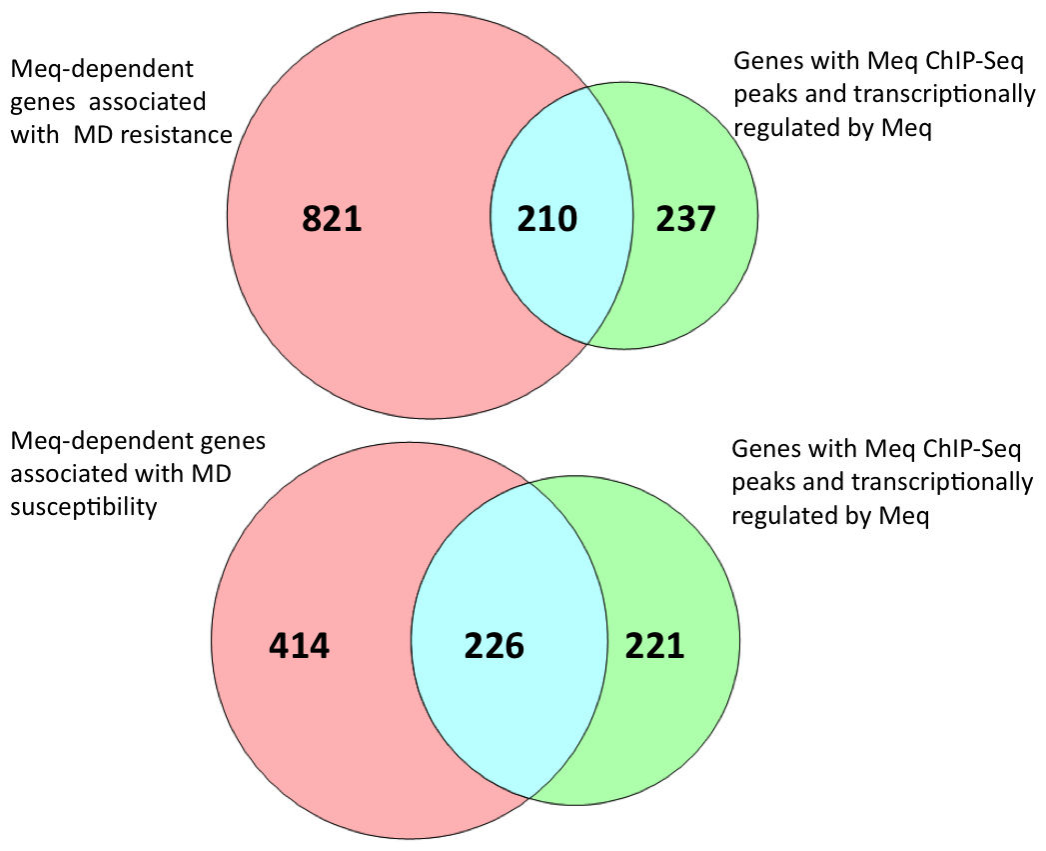
Meq-dependent genes identified through integrated analysis. Representation of overlap between Meq-dependent genes involved in MD resistance and genes with Meq ChIP-Seq peaks and transcriptionally regulated by Meq.

To further interpret if genes in Meq-regulated pathways are influenced by the genetic resistance status of the host, we combined information about Meq-bound and regulated [19] with the list of DEGs from lines 6 and 7 at a significance level of P<0.01. Interestingly, about 20% of the genes from line 6 and 35% of the genes from line 7 overlapped with Meq-bound and regulated genes previously identified *in vitro* (Figure 4).

### GO categorization of Meq-dependent gene list in CEF from MD resistant or susceptible chickens

The DEGs of Md5-infected and Md5**∆**Meq-infected groups in each of lines 6 and 7 were classified into different functional GO categories. Each of the putative genes associated with MD resistance or susceptibility were assigned to molecular function categories as designated by the GO database. Based on functional annotation clustering using the highest classification stringency, there were 53 and 44 clusters in DEGs related to MD resistance and susceptibility, respectively.

**Figure 5:**
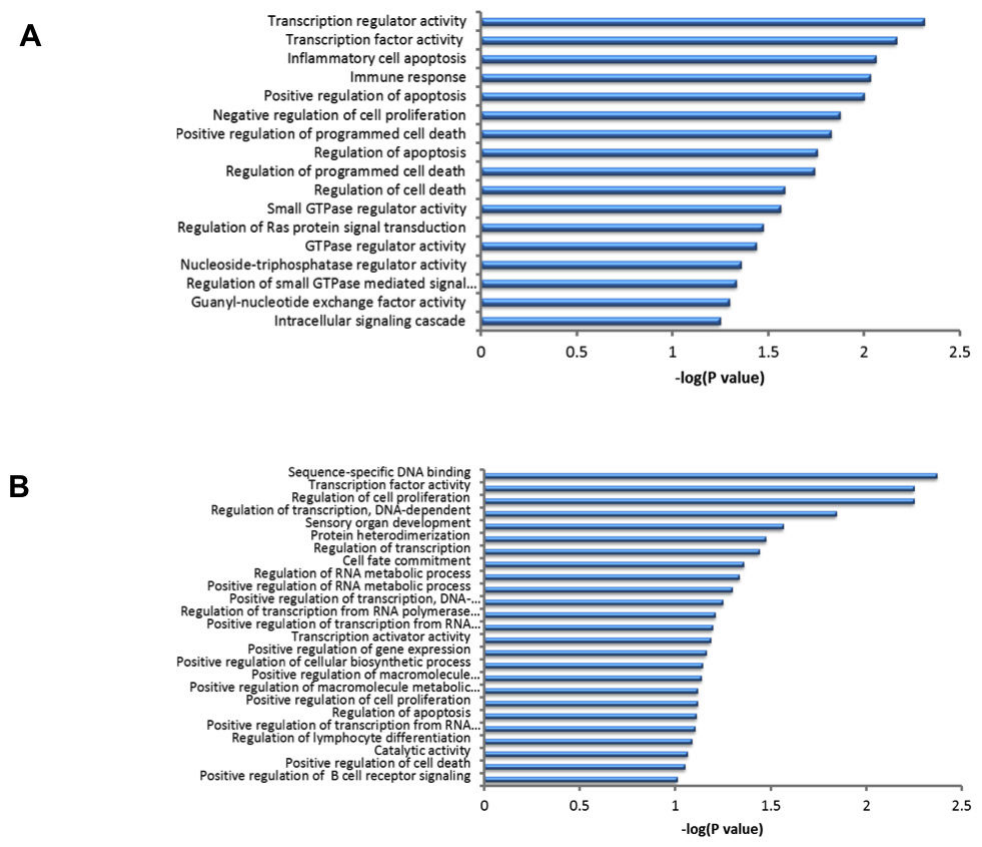
Significant molecular functions associated with genes dependent on Meq expression. The significant molecular functions are given identified through IPA based on the significantly expressed genes that were Meq-dependent and involved in MD resistance (A) and MD susceptibility (B). *P*<0.05 and FDR<0.05 were used as thresholds.

The most significant GO categories of differentially-expressed Meq-dependent genes associated with MD resistance with enrichment cutoff set to P<0.05 (Figure 5A) were transcription regulator activity, inflammatory cell apoptosis, immune response, positive regulation of apoptosis, and negative regulation of cell proliferation whereas differentially-expressed Meq-dependent genes associated with MD susceptibility (Figure 5B) were sequence-specific DNA binding, regulation of cell proliferation, DNA-dependent regulation of transcription, and protein heterodimerization. The significant categories that were common to both line 6 and line 7 Meq-dependent genes were transcription activator activity and transporter activity.

**Figure 6:**
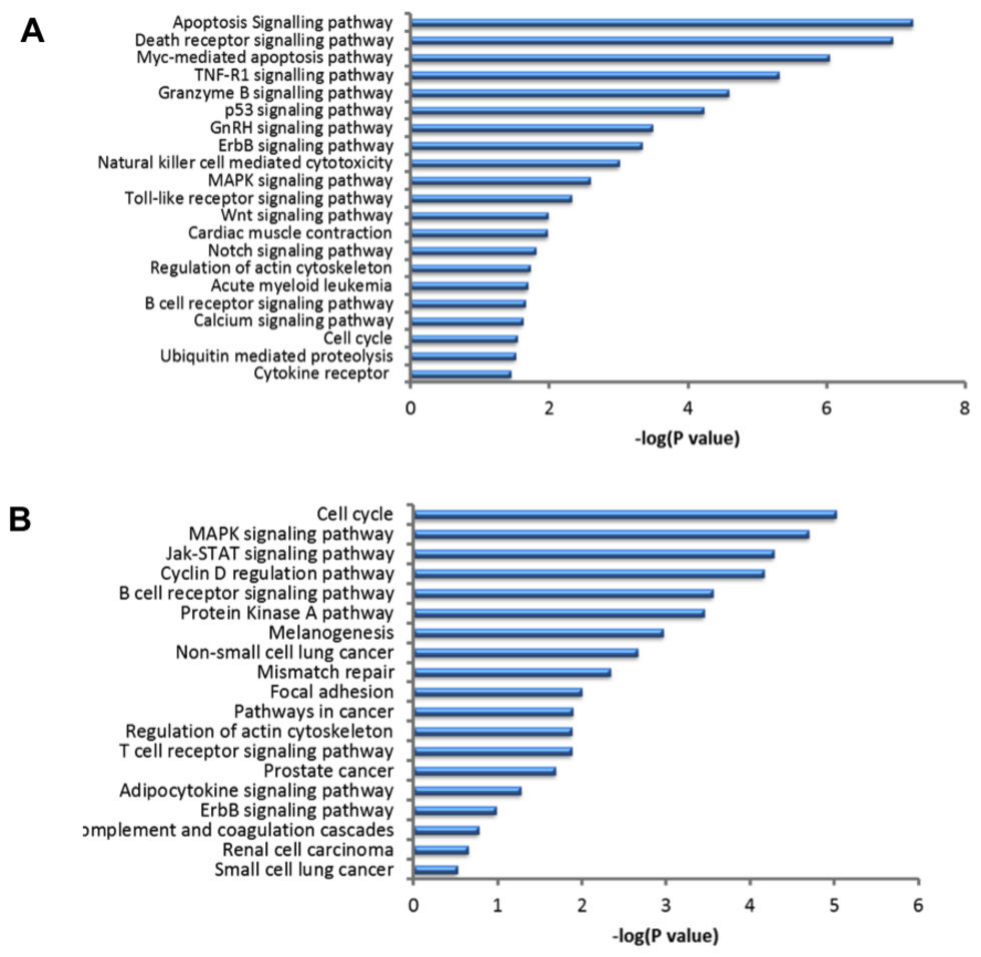
Significant pathways associated with genes dependent on Meq expression. IPA pathway analysis for significantly expressed genes that are Meq-dependent and involved in MD resistance (A) and MD susceptibility (B). *P*<0.05 and FDR<0.05 were used as thresholds to select significant canonical pathways.

To further examine the biological pathways that are altered during the host response to MDV infection in the presence of Meq, the DEGs associated with MD resistance and susceptibility were analyzed. The DEGs associated with MD resistance that were Meq-dependent were significantly associated (P<0.05) with 21 canonical pathways (Figure 6A). Some of the top pathways included apoptosis, death receptor signaling pathway, and Myc-mediated apoptosis, which plays an important role for controlled cell death. Genes involved in cell death were up-regulated and genes involved in cell maintenance were down-regulated in this category. The pathways involved in DEGs associated with MD resistance that were not dependent on Meq included DNA replication pathway, tight junction, and VEGF signaling pathway. It is interesting to note that most of Meq-dependent pathways were unique and not expressed in the group of DEGs not dependent on Meq.

Similar pathway analyses were performed on the DEGs associated with MD susceptibility that were Meq-dependent (19 significant canonical pathways; Figure 6B) and Meq-independent groups. Some of the top pathways with DEGs associated in MD susceptibility that were Meq-dependent group included cell cycle regulation, MAPK signaling pathway, and Jak-STAT signaling pathway. The pathways involved in DEGs associated in MD susceptibility that were not dependent on Meq included VEGF signaling pathway, DNA replication pathway, Hedgehog signaling pathway, mismatch repair, and insulin signaling pathway. Of these analyses, there were three pathways that were common between the two groups.

### Allele-specific binding bias of Meq at SNPs between line 6 and line 7

We have previously identified SNPs exhibiting allele-specific expression (ASE) in response to MDV infection using F_1_ birds derived by intermating lines 6 and 7 [20]. However, the mechanism for the allelic transcriptional variation in response to MDV infection is unknown. Thus, we explored whether there might be association of SNPs in the promoters of specific genes with variation in Meq-dependent binding. Specifically, we screened for genes that (1) are directly regulated by Meq, (2) exhibit ASE in response to MDV infection, and (3) have a SNP between lines 6 and 7 in the Meq-binding motif. A total of 24 candidate genes exhibited polymorphisms between the two lines in their Meq-binding motifs (e.g., TGACTCA or CACACAGC) located in their proximal promoter region (Table 2). Interestingly, this analysis revealed genes in the MAPK kinase signaling pathway (e.g., BRAF and GPS1), contributing to cell cycle regulation (e.g., FANCA, NDC80, and RB1), or coding for transcription factor subunits (e.g., AP1M1 and OPTN), all of which are known to be connected to response to virus infection and members of pathways previously implicated in our prior studies [19,20].

**Table 2:** Genes directly regulated by Meq that exhibit allele-specific expression (ASE) in response to MDV infection and have SNPs in the Meq-binding motif.

Ensembl Gene ID	Gene Symbol	Gene Name	Motifs	
ENSGALG00000009474	ACTN1	alpha-actinin-1	AACACACAT	
ENSGALG00000003959	AP1M1	AP-1 complex subunit mu-1	TGACTCA	
ENSGALG00000012865	BRAF	serine/threonine-protein kinase B-raf	CACACACA	
ENSGALG00000002495	CHCHD2	coiled-coil-helix-coiled-coil-helix domain-containing protein 2, mitochondrial	CACACACA	
ENSGALG00000019514	CNDP2	cytosolic non-specific dipeptidase	TGACTCA	
ENSGALG00000008035	COG5	conserved oligomeric Golgi complex subunit 5	TGACTCA	
ENSGALG00000011020	CYCS	cytochrome c	CACACAGC	
ENSGALG00000000516	FANCA	Fanconi anemia, complementation group A	CACACACA	
ENSGALG00000008169	GDI2	rab GDP dissociation inhibitor beta	CACACACA	
ENSGALG00000002827	GPS1	COP9 signalosome complex subunit 1	CACACAGC	
ENSGALG00000008858	MDH1	malate dehydrogenase, cytoplasmic	CACACAGC	
ENSGALG00000011377	METAP2	methionine aminopeptidase 2	CACACACA	
ENSGALG00000014801	NDC80	kinetochore protein NDC80 homolog	CACACACA	
ENSGALG00000013738	OPTN	optineurin	TGACTCA	
ENSGALG00000016997	RB1	retinoblastoma-associated protein	CACACACA	
ENSGALG00000012827	RIPK1	receptor-interacting serine/threonine-protein kinase 1	TGACTCA	
ENSGALG00000022808	RRP1B	ribosomal RNA processing protein 1 homolog B	CACACAGC	
ENSGALG00000010414	SEC 24B	protein transport protein Sec24B	CACACAGC	
ENSGALG00000009728	SLC25A13	calcium-binding mitochondrial carrier protein Aralar2	AACACACAC	
ENSGALG00000015749	ST3GAL5	lactosylceramide alpha-2,3-sialyltransferase	GACACACAC	
ENSGALG00000005535	TCOF1	Treacher Collins-Franceschetti syndrome 1	CACACACA	
ENSGALG00000007001	TLR4	toll-like receptor 4 precursor	CACACACA	
ENSGALG00000008076	TMEM164	transmembrane protein 164	CACACACA	
	ENSGALG00000011770	UMPS	uridine 5,-monophosphate synthase	AACACACAT

## Discussion

In recent years, unpredictable and spontaneous vaccine breaks resulting in devastating losses to poultry farms [1], have further necessitated the need to explore alternate strategies for MD prevention. Selection for increased genetic resistance to MD is a control strategy that has proven to work and can augment MD vaccine protection [21]. Over the years, several attempts have been made to identify candidate genes that determine genetic resistance to MD. Based on a variety of genetic and genomic strategies, these studies have shown various factors underlying the mechanism of resistance and susceptibility [5,8,12,21-24]. It is imperative to further understand the underlying mechanisms of MDV pathogenesis and further examine how this impacts genetic resistance. Being a complex disease, variability in a single gene cannot explain the basis for genetic resistance by itself. Although some previous studies have examined differential gene expression patterns after exposure to MDV in MD resistant and susceptible lines of chicken, there are no studies that have explored the role of Meq in determining genetic basis for resistance. Among several viral genes, null mutants for Meq showed no oncogenicity while knock out mutants of other viral genes like pp38 and ICP4 only resulted in attenuated virulence [25. 26]. Given the importance of Meq, we attempted to provide insights into the molecular mechanisms of MD resistance in the presence of Meq in response to MDV infection.

Through selective breeding and identification of phenotypic variation with respect to MD incidence, genetically resistant and susceptible lines of chicken have been developed [5]. One of the major contributors to resistance is variable MHC haplotypes, as evidenced by differential MHC haplotypes responsible for phenotypic variation in lines N and P [27]. However, there are numerous other factors that are non-MHC dependent which play an important role in genetic resistance to MD. Lines 6 and 7 share the same MHC haplotype but vary markedly in their susceptibilities to MD [3,28]. Hence we used this model to unravel non-MHC related basis for variable susceptibility to MD. Moreover, our study adds an essential aspect that has not previously been explored by using MDV constructs with and without Meq, its principal oncogene.

Further comparisons of results from our previous findings on genome-wide regulatory network of Meq [19] should provide additional confidence in the declared list of genes influencing resistance and susceptibility. This helps us to determine if genes in Meq-regulated pathways are influenced by the genetic resistance status of the host. We found that more than 20% of genes from line 6 and 35% of genes from line 7 were overlapping with genes that had binding sites for Meq and were transcriptionally regulated by Meq.

We hypothesized that the Meq-dependent differentially expressed genes would be involved in the downstream molecular pathways that might play an important for maintaining MD resistance. In order to identify the types of specific molecular functions of these genes, the Meq-dependent differentially expressed genes were annotated using the GO. Each gene was assigned to Molecular Function and Biological Process categories as designated by the GO database. The molecular functions and the biological process were significantly different between MD resistant and susceptible lines. The resistant line was enriched for positive regulation of cell death whereas the susceptible cell line was enriched for regulation of cell proliferation. Further analyzing the cellular pathways involved, we found that apoptosis signaling and death receptor pathways were among the significantly represented pathways. Specifically, we found an up-regulation of caspases 3, 6, and 8. Caspase 3 is an executioner caspase that is activated by both extrinsic and mitochondrial intrinsic pathways of apoptosis. Caspase 3 is activated by caspase 8 and activated caspase 3 can in turn activate other caspases like 6 and 7, which eventually leads to cell death [29]. We also noted a down-regulation of inhibitors of apoptosis like BIRC2 and antiapoptotic proteins like Bcl-2 and Bcl-X_L_. Interestingly, we have shown in a previous study that most of these genes have Meq-binding sites and are transcriptionally regulated by Meq [19]. The up-regulation of these caspases and down-regulation of anti-apoptotic factors could be one of the determinants of MD resistance in line 6. We found that mitogenic signals like MAPK signaling and regulation of cell cycle involving cyclin D were overrepresented in the line 7 transcriptome. We have previously shown that one of the major cellular pathways that Meq targets to induce transformation is the MAPK signaling pathway. Specifically, we have shown that Meq up-regulates mitogenic signals like MEK1, MEK2 and Ras, which drive the cells towards proliferation. Also, Meq down-regulates inhibitory signals like phosphatases that limit the activation of MAPK signaling. In the present study, we found that at least some of these genes are associated in differential MDV resistance/susceptibility. In the MD resistant chicken line, there was a down-regulation of MAP2K2 (MEK2) in Md5-infected group compared to Md5ΔMeq-infected group. This highlights the role of Meq in modulating MAPK signaling and how down-regulation of mitogenic signals may be associated in genetic resistance to MD. In contrast, we found an up-regulation of Ras, another mitogenic signal in line 7 infected with Md5 compared to the Md5ΔMeq-infected group, raising the possibility that up-regulation of mitogenic signals underlies genetic susceptibility to MD. These results suggest that the genes related to cell death and proliferation is one of the major determinants of genetic variability in resistance/susceptibility to MD. In one of our previous studies using RNA sequencing analysis, we have shown that apoptotic signaling is significantly overrepresented in spleens of birds infected with MDV [30].

Our group has previously demonstrated that Jak-STAT is an important cellular pathway involved in genetic basis of MD resistance [20,30]. Similarly, we also noted that genes in Jak-STAT pathway are transcriptionally up-regulated in susceptible line 7 and not in the resistant line 6. Activation of Jak-STAT pathway results in nuclear translocation of activated STAT dimer resulting in transcription of genes involved in cell survival and proliferation [31]. In addition to MAPK pathway, this could be another mitogenic signaling pathway that is involved in transformation by Meq.

We have previously found a subset of transcription factor binding sites from ChIP-seq analysis. Further comparison with our previous findings on the ASE between lines 6 and 7 provides additional confidence in the list of genes regulated by Meq that exhibit polymorphisms at the proximal promoter region and allele-specific gene expression. Additionally, we also found that many allele-specific binding shows a bias between line 6 and line 7, which potentially represents the functional distinction between MD resistance and susceptibility. If planned experiments show an association between these SNPs and MD incidence, then it would validate the importance of Meq-regulated transcription in MD genetic resistance.

We also found down-regulation of IRG1, a putative proapoptotic factor which was recently described as a candidate susceptibility gene for MD [11] in line 7 but no expression in line 6. The fact that we noted this transcriptional response in line 7 infected with Md5 and not Md5**∆**Meq-infected group raises an important and novel corollary that Meq has a role in regulating IRG1 expression. Further, we also noted transcriptional regulation of other genes (STAT1, MyD88, IFN-γ) proposed in the biological interaction network analysis of IRG1 in the previous study. In addition to corroborating the results from that study, this further underscores the role of Meq in modulating apoptotic signaling as a determinant of MD susceptibility.

Admittedly, CEFs are not the natural target cells for MDV. However, it is worth noting transcript profiling using spleens and other organs suffers from the potential confounding influence of differences in cellular composition and reagents to properly quantify each cell subtype are limiting in chicken. Thus, the high degree of overlap between our current results, which utilized cultured cells from defined chicken lines, and those from our other studies that used actual birds [11,12,20,30] for the identification of critical pathways suggests that CEF are reasonable models. This agreement also suggests an important contribution of innate immunity towards MD genetic resistance. Furthermore, as cells and viruses can be more easily manipulated and monitored compared to actual MDV challenges of live birds, this suggests that more precise experiments or hypotheses should be first explored *in vitro* prior to animal studies, especially when one wishes to screen for the direct influences of particular viral proteins like Meq.

In conclusion, we have made significant insights on the different sets of genes and pathways that interact to modulate MD resistance and susceptibility. Taken together, our findings add to the current understanding of the mechanism behind Meq-induced responses that lead to MD resistance or susceptibility. The overlap between SNPs from ASE and ChIP-seq data show that allele-specific Meq binding at many of these SNPs could allow a functional distinction between line 6 and line 7. We have confirmed that allele-specific binding biases by Meq tend to be one of the underlying genetic mechanisms for line 6 and line 7 alleles. In addition, this study forms the basis for selection of candidate genes that might be associated in genetic resistance to MD.

## References

[1] Witter RL (1997) Increased virulence of Marek's disease virus field isolates. Avian Dis 41: 149-163. doi:10.2307/1592455. PubMed: 9087332. 9087332

[2] Gimeno IM (2008) Marek's disease vaccines: a solution for today but a worry for tomorrow? Vaccine 26 (Suppl 3): C31-C41. doi:10.1016/j.vaccine.2008.04.009. PubMed: 18773529. 18773529

[3] Stone H (1975) Use of highly inbred chickens in research USDA. Technical Bulletin 1514.

[4] Bacon LD, Hunt HD, Cheng HH (2000) A review of the development of chicken lines to resolve genes determining resistance to diseases. Poult Sci 79: 1082-1093. PubMed: 10947175. 10947175 10.1093/ps/79.8.1082

[5] Bumstead N, Kaufman J (2004) Genetic resistance to Marek's disease. In: Davison F Nair V Marek's Disease: An Evolving Problem. San Diego: Elsevier - Academic Press . pp. 112-123

[6] Briles WE, Stone HA, Cole RK (1977) Marek's disease: Effects of B histocompatibility alloalleleles in resistant and susceptible chicken lines. Science 195: 193-195. doi:10.1126/science.831269. PubMed: 831269. 831269

[7] Cheng HH, Kaiser P, Lamont SJ (2013) Integrated genomics approaches to enhance genetic resistance in chickens. Ann Rev Anim Vet Biosci 1: 239-260 10.1146/annurev-animal-031412-103701 25387019

[8] Liu HC, Kung HJ, Fulton JE, Morgan RW, Cheng HH (2001) Growth hormone interacts with the Marek's disease virus SORF2 protein and is associated with disease resistance in chicken. Proc Natl Acad Sci U_S_A 98: 9203-9208. doi:10.1073/pnas.161466898. PubMed: 11470922. 11470922 PMC55398

[9] Liu HC, Niikura M, Fulton JE, Cheng HH (2003) Identification of chicken lymphocyte antigen 6 complex locus E (LY6E alias SCA2) as a putative Marek's disease resistance gene via a virus-host protein interaction screen. Cytogenet Genome Res 102: 304-308. doi:10.1159/000075767. PubMed: 14970721. 14970721

[10] Niikura M, Kim T, Hunt HD, Burnside J, Morgan RW, Dodgson JB, Cheng HH (2007) Marek's disease virus up-regulates major histocompatibility complex class II cell surface expression in infected cells. Virology 359: 212-219. doi:10.1016/j.virol.2006.09.010. PubMed: 17028059. 17028059

[11] Smith J, Sadeyen JR, Paton IR, Hocking PM, Salmon N et al. (2011) Systems analysis of immune responses in Marek's disease virus-infected chickens identifies a gene involved in susceptibility and highlights a possible novel pathogenicity mechanism. J Virol 85: 11146-11158. doi:10.1128/JVI.05499-11. PubMed: 21865384. 21865384 PMC3194948

[12] Yu Y, Luo J, Mitra A, Chang S, Tian F et al. (2011) Temporal transcriptome changes induced by MDV in Marek's disease-resistant and -susceptible inbred chickens. BMC Genomics 12: 501. doi:10.1186/1471-2164-12-501. PubMed: 21992110. 21992110 PMC3269463

[13] Burgess SC, Basaran BH, Davison TF (2001) Resistance to Marek's disease herpesvirus-induced lymphoma is multiphasic and dependent on host genotype. Vet Pathol 38: 129-142. doi:10.1354/vp.38-2-129. PubMed: 11280369. 11280369

[14] Sarson AJ, Parvizi P, Lepp D, Quinton M, Sharif S (2008) Transcriptional analysis of host responses to Marek's disease virus infection in genetically resistant and susceptible chickens. Anim Genet 39: 232-240. doi:10.1111/j.1365-2052.2008.01710.x. PubMed: 18371127. 18371127

[15] Niikura M, Kim T, Silva RF, Dodgson J, Cheng HH (2011) Virulent Marek's disease virus generated from infectious bacterial artificial chromosome clones with complete DNA sequence and the implication of viral genetic homogeneity in pathogenesis. J Gen Virol 92: 598-607. doi:10.1099/vir.0.026864-0. PubMed: 21123546. 21123546

[16] Silva RF, Dunn JR, Cheng HH, Niikura M (2010) A MEQ-deleted Marek's disease virus cloned as a bacterial artificial chromosome is a highly efficacious vaccine. Avian Dis 54: 862-869. doi:10.1637/9048-090409-Reg.1. PubMed: 20608531. 20608531

[17] Wettenhall JM, Simpson KM, Satterley K, Smyth GK (2006) affylmGUI: a graphical user interface for linear modeling of single channel microarray data. Bioinformatics 22: 897-899. doi:10.1093/bioinformatics/btl025. PubMed: 16455752. 16455752

[18] Livak KH, Schmittgen TD (2001) Analysis of relative gene expression data using real-time quantitative PCR and the. p. 2-CT method. Methods 25: 402-408. 10.1006/meth.2001.1262 11846609

[19] Subramaniam S, Johnston J, Preeyanon L, Brown CT, Kung HJ, Cheng HH (2013) Integrated analyses of genome-wide DNA occupancy and expression profiling identify key genes and pathways involved in cellular transformation by Marek's disease oncoprotein Meq. J Virol 87: 9016-9029. doi:10.1128/JVI.01163-13. PubMed: 23740999. 23740999 PMC3754031

[20] Perumbakkam S, Muir WM, Black-Pyrkosz A, Okimoto R, Cheng HH (2013) Comparison and contrast of genes and biological pathways responding to Marek's disease virus infection using allele-specific expression and differential expression in broiler and layer chickens. BMC Genomics 14: 64. doi:10.1186/1471-2164-14-64. PubMed: 23363372. 23363372 PMC3599046

[21] Bacon LD, Hunt HD, Cheng HH (2001) Genetic resistance to Marek's disease. Curr Top Microbiol Immunol 255: 121-141. doi:10.1007/978-3-642-56863-3_5. PubMed: 11217420. 11217420

[22] Vallejo RL, Bacon LD, Liu HC, Witter RL, Groenen MA et al. (1998) Genetic mapping of quantitative trait loci affecting susceptibility to Marek's disease virus induced tumors in F2 intercross chickens. Genetics 148: 349-360. PubMed: 9475745. 9475745 10.1093/genetics/148.1.349 PMC1459797

[23] McElroy JP, Dekkers JC, Fulton JE, O'Sullivan NP, Soller M et al. (2005) Microsatellite markers associated with resistance to Marek's disease in commercial layer chickens. Poult Sci 84: 1678-1688. PubMed: 16463964. 16463964 10.1093/ps/84.11.1678

[24] Cheng H, Niikura M, Kim T, Mao W, MacLea KS et al. (2008) Using integrative genomics to elucidate genetic resistance to Marek's disease in chickens. Dev Biol 132: 365-372. PubMed: 18817328. 10.1159/000317187 18817328

[25] Jones D, Lee L, Liu JL, Kung HJ, Tillotson JK (1992) Marek disease virus encodes a basic-leucine zipper gene resembling the fos/jun oncogenes that is highly expressed in lymphoblastoid tumors. Proc Natl Acad Sci U_S_A 89: 4042-4046. doi:10.1073/pnas.89.9.4042. PubMed: 1315048. 1315048 PMC525628

[26] Liu JL, Lee LF, Ye Y, Qian Z, Kung HJ (1997) Nucleolar and nuclear localization properties of a herpesvirus bZIP oncoprotein MEQ. J Virol 71: 3188-3196. PubMed: 9060682. 9060682 10.1128/jvi.71.4.3188-3196.1997 PMC191451

[27] Cole RK (1968) Studies on genetic resistance to Marek's disease. Avian Dis 12: 9-28. PubMed: 5643702. 5643702

[28] Longenecker BM, Pazderka F, Gavora JS, Spencer JL, Stephens EA et al. (1977) Role of the major histocompatibility complex in resistance to Marek's disease: restriction of the growth of JMV-MD tumor cells in genetically resistant birds. Adv Exp Med Biol 88: 287-298. PubMed: 21547. 21547 10.1007/978-1-4613-4169-7_27

[29] Estaquier J, Vallette F, Vayssiere JL, Mignotte B (2012) The mitochondrial pathways of apoptosis. Adv Exp Med Biol 942: 157-183. doi:10.1007/978-94-007-2869-1_7. PubMed: 22399422. 22399422

[30] MacEachern S, Muir WM, Crosby SD, Cheng HH (2011) Genome-wide identification and quantification of cis- and trans-regulated genes responding to Marek's disease virus infection via analysis of allele-specific expression. Front Genet 2: 113 PubMed: 22303407. 22303407 10.3389/fgene.2011.00113 PMC3268648

[31] Boudny V, Kovarik J (2002) JAK/STAT signaling pathways and cancer Janus kinases/signal transducers and activators of transcription. Neoplasma 49: 349-355. PubMed: 12584581. 12584581

